# Modifications of hemoglobin and myoglobin by Maillard reaction products (MRPs)

**DOI:** 10.1371/journal.pone.0188095

**Published:** 2017-11-14

**Authors:** Aristos Ioannou, Constantinos Varotsis

**Affiliations:** Department of Environmental Science and Technology, Cyprus University of Technology, Limassol, Cyprus; University of Colorado Denver School of Medicine, UNITED STATES

## Abstract

High performance liquid chromatography (HPLC) coupled with a Fraction Collector was employed to isolate Maillard reaction products (MRPs) formed in model systems comprising of asparagine and monosaccharides in the 60–180°C range. The primary MRP which is detected at 60°C is important for Acrylamide content and color/aroma development in foods and also in the field of food biotechnology for controlling the extent of the Maillard reaction with temperature. The discrete fractions of the reaction products were reacted with Hemoglobin (Hb) and Myoglobin (Mb) at physiological conditions and the reaction adducts were monitored by UV-vis and Attenuated Total Reflection-Fourier transform infrared (FTIR) spectrophotometry. The UV-vis kinetic profiles revealed the formation of a Soret transition characteristic of a low-spin six-coordinated species and the ATR-FTIR spectrum of the Hb-MRP and Mb-MRP fractions showed modifications in the protein Amide I and II vibrations. The UV-vis and the FTIR spectra of the Hb-MRPs indicate that the six-coordinated species is a hemichrome in which the distal E7 Histidine is coordinated to the heme Fe and blocks irreversibly the ligand binding site. Although the Mb-MRPs complex is a six-coordinated species, the 1608 cm^-1^ FTIR band characteristic of a hemichrome was not observed.

## Introduction

**Fig 1:**
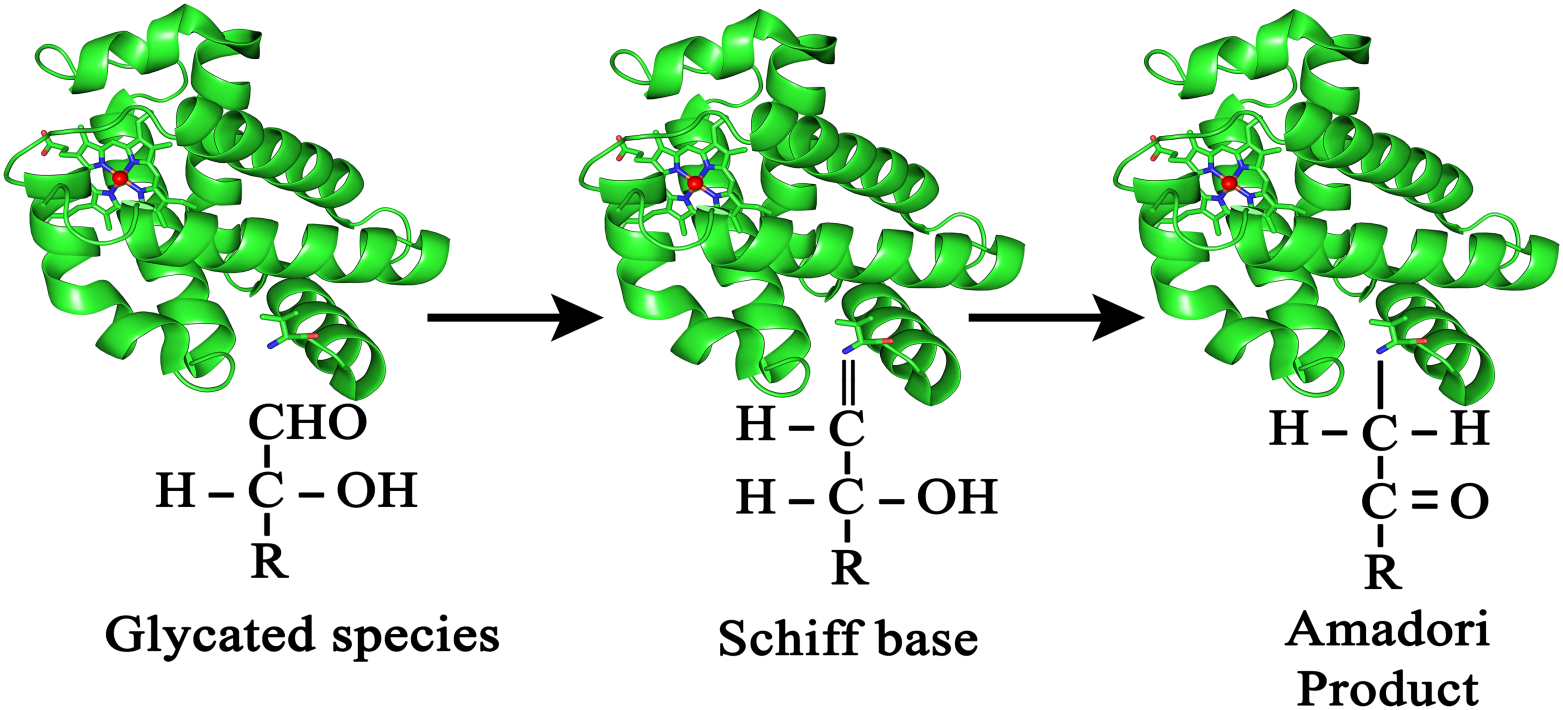
The glycation process in hemoglobin as related to protein structural modifications by glycated species.

The glycation process as illustrated by Maillard reactions and its health consequences have gained considerable attention and have been studied extensively in recent years [1–9]. Glycation processes cause the pathology in diabetes principally by the non-enzymatic modification of proteins by glucose and other products of glucose metabolism. The glycation reaction’s kinetics are heightened by elevated and prolonged exposure to glucose and other glycated species, which in turn leads to the chronic health problems. The classical example is that of the post-translational modification of hemoglobin occurring from the covalently bound intermediate arising from the interaction of the electrophilic glucose groups with the nucleophilic primary amino-groups of protein amino-acid residues. Hemoglobin advanced glycation end products (Hb-AGEs) are formed when the initial Schiff bases typically undergoes an Amadori rearrangement resulting in the formation a fructosamin (ketosamin) [10] (Fig 1).

Glycated hemoglobin (Hb) is an effective index of long-term blood glucose level and has been widely used in the diagnosis of diabetes mellitus. Glycated Hb comprises HbA1 (Amadori product) and other Hb variants and adducts [11]. HbA1c which is the major component of HbA1 is formed by a non-enzymatic irreversible process in which the aldehyde group of glucose combines with the amino-terminal valine of the β-chain of Hb [12]. More broadly, the terms glycated hemoglobin or glycohemoglobin (GHb, GlcHb) refer to the full range of glycated hemoglobins, including those containing glycated valine and/or lysine residues. The major hemoglobin glycation sites *in vitro* and *in vivo* include β-Lys-66 and β-Lys-120 [13]. The presence of an AGE-modification alters or destroys enzymatic activity, one example being methylglyoxal-modified serum albumin [14].

Hemichromes are formed when hemoglobin undergoes conformational changes resulting in the formation of a six-coordinated Fe^3+^ low-spin His-Fe-His species [15]. Hemoglobin A in humans can form hemichromes even under physiological conditions as a result of pH and temperature alterations, and ιn the autoxidation of oxyHb [15]. Hemichromes are mainly produced by partially denatured hemoglobin and thought to be precursors of Hb denaturation processes, such as unfolding, precipitation, and heme dissociation. In tetrameric α2β2 Hb it is always thought that oxidized forms adopt either aquo-met or hydroxy-met states, according on the pH of their medium. However, it was recently shown that alternate forms like bis-histidyl hemichrome states could be compatible with folded structures [16]. The crystal structure of Hb in which a partial hemichrome was formed has been reported [17].

In the Maillard reactions a cascade of events each with a discrete role can affect the product composition and generate distinct colors and aromas. One of the factors with instructive significance is temperature and for this reason we have investigate the reaction in the 60–180°C range. Many of the studies on protein glycation deal with identification of the glycated protein sites by LC-MS. In this work, we have monitored the reactions of the isolated MRPs products which are originated from the reactions of 1) asparagine with glucose and 2) asparagine with fructose and were separated and isolated by an HPLC component system coupled to a fraction collector in the 60–180°C range and found that the primary reaction product is formed at 60°C. Because the Maillard reaction is a cascade of consecutive and parallel reaction steps, it is important for the food biotechnology industry to be able to control the extent of the Maillard reaction with temperature. All reaction products were reacted with Hb and Mb. Our results demonstrate the formation of hemichromes in the reactions of the individual MRPs products originated from the Asn/Gluc and Asn/Fruc reactions with Hb, as indicated by 1) the UV-vis data for the formation of a six-coordinate species and 2) the observation of the 1608 cm^-1^ protein Hb FTIR band. On the other hand, although Mb has a number of ε-amino groups which could be glycated as similarly occurring in Hb and the UV-vis difference spectra show peaks/troughs at 425/407 nm with a zero-crossing at 420 nm which is characteristic of a six-coordinated species, the 1608 cm^-1^ FTIR band was not observed in none of the Mb-MRPs. Of note is a 6 cm^-1^ downshift in both the Amide I and II bands that demonstrate conformational changes in all of the Mb-MRPs.

## Materials and methods

### Sample preparation

Equimolar solutions of glucose or fructose and asparagine (0.2 M) were prepared in phosphate buffer (50 mM) and the pH was adjusted to 8.0. Samples (10 ml) were heated in closed screw-capped tubes at 60, 80, 100 and 180°C in a heating oven (Memmert, Germany) for 2 hours. For HPLC analysis the samples were diluted hundred-fold.

### Spectroscopic monitoring of hemoglobin with added MRPs

Hemoglobin and myoglobin (1 mM) were dissolved in 100 mM potassium phosphate buffer (pH 8.0) at 25°C. The native protein solutions (oxidized form) were used as the blank sample in the spectrophotometric cell. Hemoglobin and myoglobin were diluted hundred-fold and immediately mixed with each individual LC fraction (2:1 v/v) from the reaction between asparagine and glucose or fructose. Immediately upon mixing the protein and fraction solutions, a time cycle program on the UV-vis spectrophotometer software was commenced. The time cycle program allows for the automated collection of sample measurements at predetermined time periods.

### HPLC-Fraction collector analysis

The customized HPLC experimental setup consisted of a Varian 218 Prepstar Solvent Delivery Module, an Agilent Manual FL-Injection Valve, an Agilent 1260 Infinity Variable Wavelength Detector (VWD) and an Agilent 440 LC Fraction Collector. Water at a flow rate of 0.5 ml/min was used isocratically as the mobile phase at room temperature. A 20 μL aliquot of sample was injected. Maillard reaction products (MPRs) were detected at 200 nm. An automated single probe Agilent Technologies 440 fraction collector was coupled to the chromatographic system. The instrumental setup and the separation of individual fractions were described in detail earlier [18]. HPLC analysis was performed on a 4.6 x 250 mm, 5 μm particle size, Zorbax SB-Aq analytical column (Agilent Technologies). Maillard reaction products (MPRs) were analyzed under aqueous conditions using a previously reported method [19]. The fraction collector employed the Agilent OpenLAB Software real-time peak detection algorithms to achieve accurate and reproducible chromatographic detection of fractions. Individual resolved elements from a multi-component sample were collected by means of a time-slice method. During the chromatographic run the fraction collector allows for collection at defined time intervals. Fractions were collected in time slices thus creating a time frame window for each chromatographic peak. This was achieved by means of a diverter valve which is switched from the waste to the collect position. The time frames for each eluting peak and the system delay time were incorporated to a time program table created by the Agilent OpenLAB Software. Mobile phase during equilibration between chromatographic runs was sent to the waste position since the valve was switched back to this position after the finish of each run. Pooling of identical fractions between runs was also performed to enhance FTIR detection.” The instrumental setup and the separation of individual fractions were described in detail earlier [18]. HPLC analysis was performed on a 4.6 x 250 mm, 5 μm particle size, Zorbax SB-Aq analytical column (Agilent Technologies). Maillard reaction products (MPRs) were analyzed under aqueous conditions using a previously reported method [19].

### UV-vis spectrophotometry

Hemoglobin and myoglobin were dissolved in 100 mM potassium phosphate buffer (pH 8.0) at 25°C. The native protein solutions (oxidized form) were used as the blank sample in the spectrophotometric cell. Once the protein solutions were used to perform the blank measurement, the fraction solutions were added to the protein solution in the cell and the first sample measurement at time 0 minutes was performed by means of a time cycle program on the UV-vis Lambda 25 Perkin Elmer spectrophotometer software. Difference spectra (reacted Hb or Mb with MRPs *minus* oxidized Hb or Mb) were collected at time intervals of 5 minutes apart for a time period of 120 minutes in the wavelength range 200–500 nm. The time cycle program allows for the automated collection of sample measurements at predetermined time periods.

### ATR-FTIR spectrophotometry

A horizontal ATR (HATR) accessory (Pike Technologies, Inc, Madison, USA) was employed fitting a Germanium ATR plate with ten internal reflections. The Horizontal ATR employs a pair of transfer optics to direct the infrared beam of the spectrometer to one end of the IR transmitting ATR crystal. A similar pair of optics directs the beam emitted from the other end of the ATR crystal to the spectrometer detector. A Tensor 27 FTIR spectrometer (Bruker, Karlsruhe, Germany) equipped with a deuterated triglycine sulfate (DTGS) detector was used for spectral acquisition. Spectra were collected in the range 1,800–800 cm^−1^ with 4 cm^−1^ resolution and 100 co-added scans each. A background spectrum was collected before each sample measurement. The software package OPUS 7.0/IR (Bruker) was used to acquire and process the FTIR spectra.

## Results and discussion

### Isolation and characterization of MRPs by HPLC-Fraction collector

**Fig 2:**
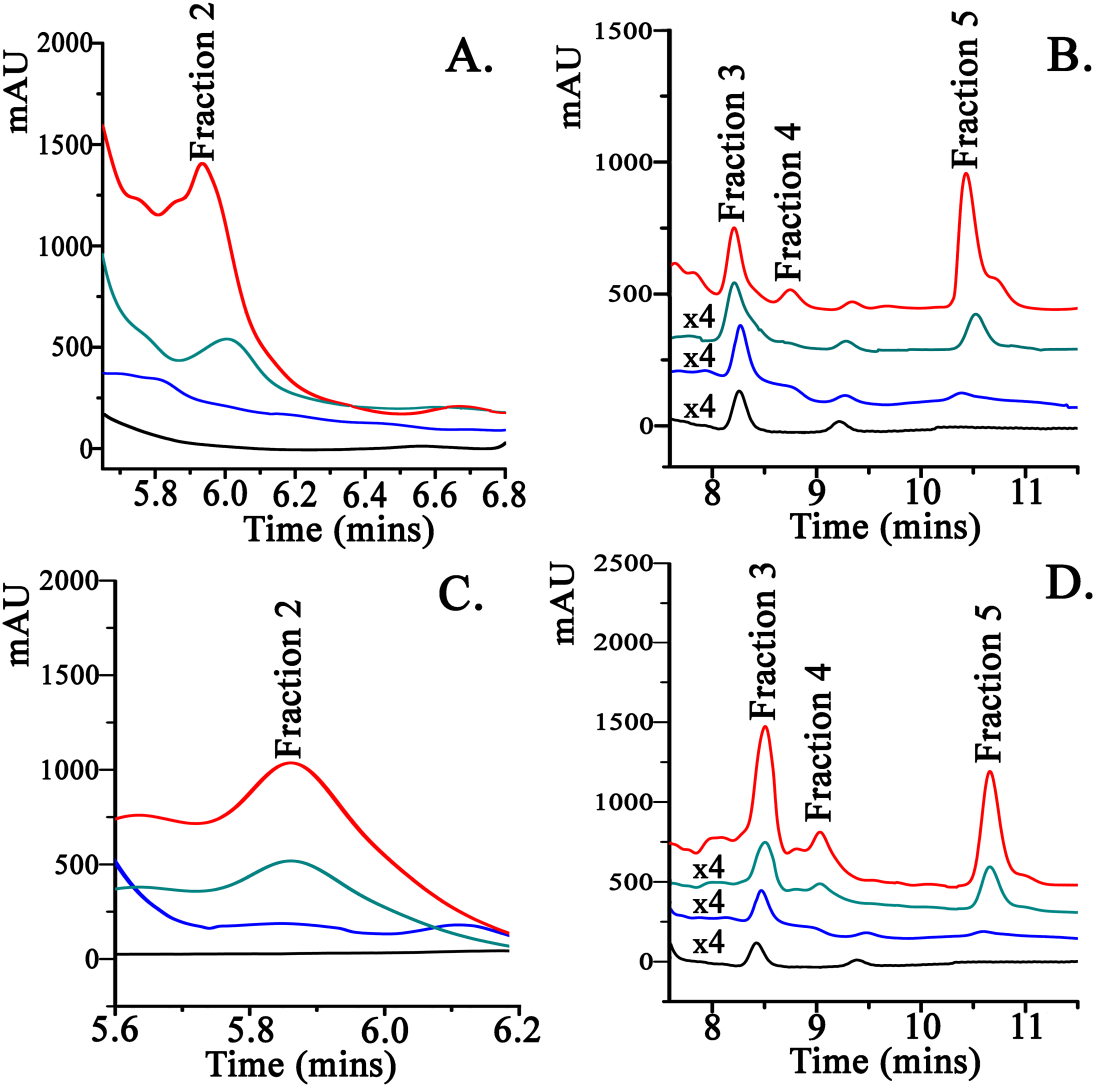
High performance liquid chromatography (HPLC) chromatograms of the reaction mixtures of asparagine and sugars (black: 60°C, blue: 80°C, cyan: 100°C, red: 180°C). A 20 μL aliquot of sample was injected and the wavelength used for detection was 200 nm. Triplicate samples were manually injected in the chromatographic system. **Panel A**: Fraction 2 from the reaction of asparagine with glucose. **Panel B**: Fractions 3–5 collected as discrete MRPs in the reaction of asparagine with glucose. **Panel C**: Fraction 2 from the reaction of asparagine with fructose. **Panel D**: Fractions 3–5 collected as discrete MRPs in the reaction of asparagine with fructose.

Glycoconjugates, such as N-glycosides and related compounds are recognized as key Maillard reaction intermediates [20]. When asparagine and glucose (Fig 2A and 2B) or fructose (Fig 2C and 2D) reacts in a 60–180°C range it is evident from the HPLC chromatograms that there is elution of several individual products in the reaction mixture (Fig 2). These are even formed at lower temperatures but their yield is significantly increased at higher temperatures. Maillard reaction products (MRPs) were eluted on the Zorbax column which can retain a high selectivity for polar molecules in aqueous phases [19]. Fraction 1 was a combined fraction encompassing a major signal at Rt = 5.7 min corresponding to the excess unreacted asparagine arising from the heated reaction mixture [18]. Fractions 2–5 corresponding to discrete MRPs were collected separately. Fractions 2, 3 and 5 have been characterized to originate from the Schiff base, the Amadori product and acrylamide, respectively [18].

All fractions displayed characteristic absorbance bands around 294 nm [18] which was previously reported as a generic marker band of Maillard reaction intermediate products [21–23]. These compounds relate to the glycation process as being aldehydes and small molecule ketones arising from the glycosylation of the asparagine molecule. UV absorbance in the range of 270–300 nm is characteristic for molecules containing carbonyl groups (n->π* transitions).

### Modifications of hemoglobin and myoglobin by MRPs

**Fig 3:**
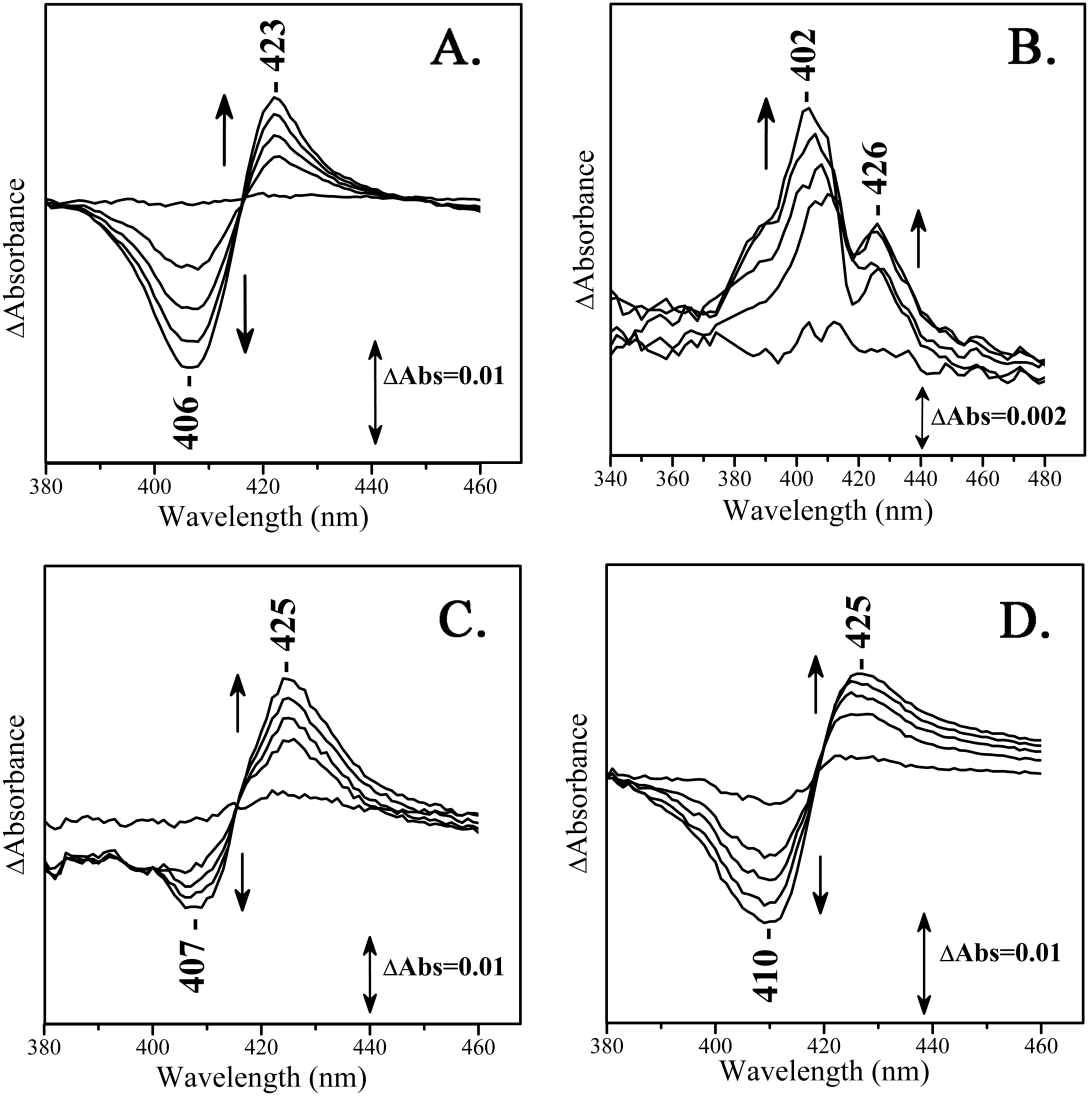
UV-vis difference spectra of Hb- and Mb-MRPs (LC fraction 2) adducts *minus* oxidized Hb or Mb. In panels A (LC fraction 2 from the reaction of Asn with Gluc) and B (LC fraction 2 from the reaction of Asn with Fruc) are the spectra of Hb-MRP adducts formed in the reaction with the LC fraction 2 and in panel C and D are the analogous spectra of Mb. Time course: 0-120mins; Spectra displayed every 30 minutes).

Fig 3A depicts the time evolution of the difference absorption spectrum of the reaction product of Hb which is formed upon the addition of the Schiff base LC fraction 2 from the reaction of asparagine and glucose at pH 8 *minus* oxidized Hb. The difference spectrum shows maxima at 423 nm and minima at 406 nm. The 423 nm transition is attributed to the formation of a six-coordinate species. It has been shown that the selection of a wavelength close to 423 nm is possible a precise measurement for the concentration of glycated hemoglobin [24]. Similar observations have been reported where the spectral difference between hemoglobin and hemichrome was monitored by the difference spectrum with a minimum at 405 nm and a maximum at 423 nm in a fatty acid-hemoglobin system [25]. The UV-vis difference spectra of Hemoglobin with added LC fraction 2 of the reaction mixture of asparagine and fructose, show a 426 nm band that resembles that at 423 nm shown in panel A and an additional transition at 402 nm (Fig 3B). Obviously, the Hb-MRPs originated from the Asn/Glu and Asn/Fruc reactions differ significantly. On the other hand, the analogous Mb-MRPs show strong similarities as indicated by the observation of a peak/trough at 425/410 nm, in both the reactions with the isolated compounds from the Asn/Glu and Asn/Fruc reactions (Fig 3C and 3D).

**Fig 4:**
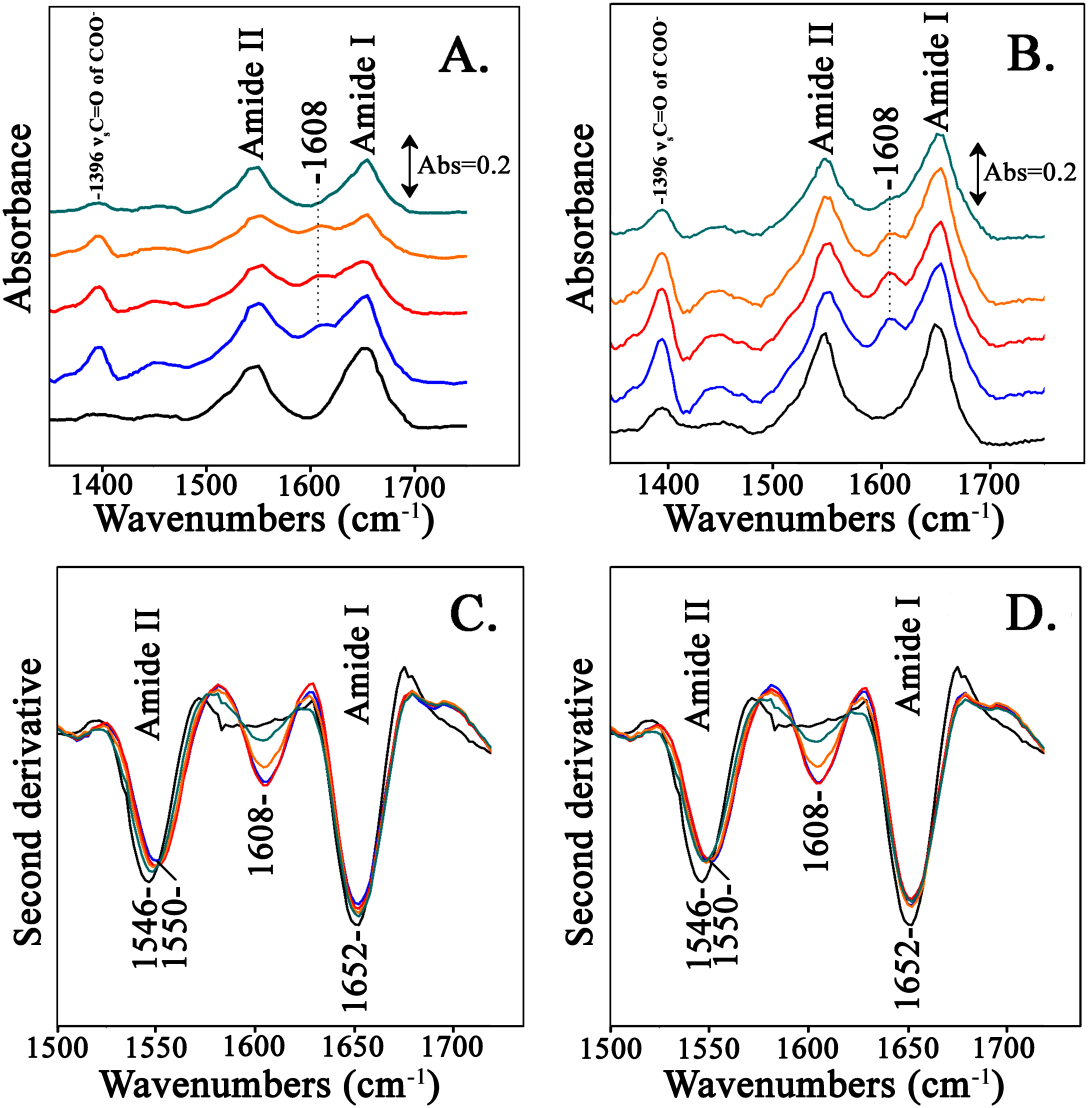
ATR-FTIR spectra of hemoglobin with added LC fractions 2–5 (Black: Hb, Blue: Hb + fraction 2, Red: Hb + fraction 3, Orange: Hb + fraction 4, Cyan: Hb + fraction 5). **Panel A**. Fractions 2–5 are from the reaction of asparagine with glucose. **Panel B**. Fractions 2–5 are from the reaction of asparagine with fructose. **Panel C**. Second derivative spectra of those presented in panel A. **Panel D**. Second derivative spectra of those presented in panel B.

Fig 4A–4D shows the FTIR spectra of the Hb-adducts at pH 8.0 formed from the reactions of Hb with the compounds from the LC Fractions 2–5. The LC fractions were also subjected to ATR-FTIR analysis. However, the LC fractions did not show any FTIR absorption bands due to their low concentration which is much lower than the detection limit of our current ATR-FTIR experimental setup. Therefore, there is no interference from the LC fractions in the ATR-FTIR spectra. In Fig 4A are the FTIR spectra of the Hb-adducts formed from the reactions of Hb with the compounds from the LC fractions 2–5 from the reaction of Asparagine with Glucose and in Fig 4B those from the reactions of LC fractions 2–5 of Asparagine with Fructose. Fig 4C shows the second derivative spectra of the spectra shown in Fig 4A and in Fig 4D are the second derivative spectra of the spectra shown in Fig 4B. Amides I and II are the major bands in the IR spectrum of a protein. Amide I absorption originates from the C = O stretching vibration (70–85%) of the amide group (coupled to in-phase bending of the N–H bond and stretching of the C–N bond), which gives rise to IR band(s) in the region between ~1600 and 1700 cm^–1^ [25–28]. Amide II originates from the N–H bending (40–60%) and C–N stretching vibrations (18–40%) [26–29]. From the ATR-FTIR spectra, the ratio of the Amide I to Amide II is slightly lower in the Hb-LC Fractions spectra compared to the initial Hb (Fig 4A and 4B) suggesting conformational changes. Another evident feature from the ATR-FTIR spectra is the observation of a new band at 1608 cm^-1^ in all spectra of the Hb-adducts. We attribute the appearance of the new 1608 cm^-1^ band to the formation of strong beta-sheet structures [30]. In a recent study probing the *α*-helix to *β*-sheet transition in fibrin, a band at 1612–1614 cm^-1^ was assigned as a marker band of nascent inter-chain *β*-sheets, consistent with protein aggregation [31]. An additional spectral feature is the increase in intensity of the 1396 cm^-1^ Hb band which corresponds to the symmetric C = O stretching vibration of COO^-^ groups. This absorption band is related to aspartic acid residues which function as important subunit contact sites. The Tyrα42⋯Aspβ99 H-bond is in the “switch” region of the α_1_β_2_ interface [32] involving the interface residues between the αC helix and the βFG corner experience large dislocations during R to T transition. An equally important hydrogen bonded pair is the Trpβ37⋯Aspα94 which comprises of the “hinge” region, in which the R–T shift is restricted to a change in orientation [32]. The above mentioned feature is particularly evident and it seems to be coupled to the appearance of the 1608 cm^-1^ band in the Hb-LC Fractions 2–4. Fraction 5 forms a very low yield compared to fractions 2–4 of the newly formed 1608 cm^-1^ band and the 1396 cm^-1^ band does not exhibit any intensity change. Similar results are obtained in the reactions of Hb with the reactions products of Asn/ Fruc (LC fractions 2–5) shown in Fig 4B. The second derivative spectra of the Hb-adducts formed from the reactions of both the Asn/Gluc and Asn/Fruc reactions shown in Fig 4C and 4D, respectively, show that the amide I vibration remains constant and small frequency changes in the amide II vibration. The presence of the 1608 cm^-1^ band is evident in both cases.

**Fig 5:**
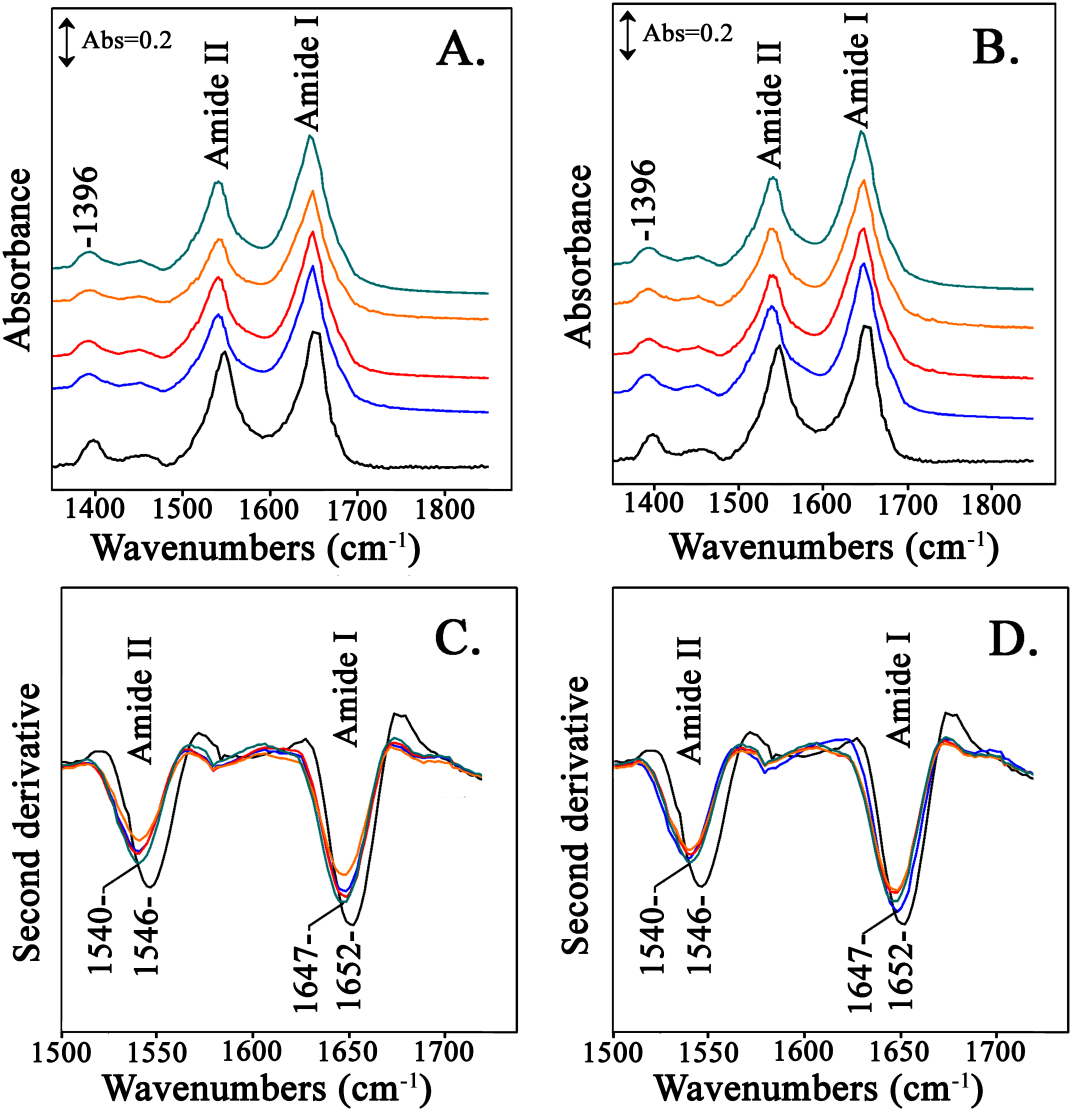
ATR-FTIR spectra of myoglobin with added LC fractions (Black: Mb, Blue: Mb + fraction 2, Red: Mb + fraction 3, Orange: Mb + fraction 4, Cyan: Mb + fraction 5). **Panel A**. Fractions 2–5 are from the reaction of asparagine with glucose. **Panel B**. Fractions 2–5 are from the reaction of asparagine with fructose. **Panel C**. Second derivative spectra of those presented in panel A. **Panel D**. Second derivative spectra of those presented in panel B.

In the case of Mb-LC Fractions spectra (Fig 5A and 5B), we do not observe the presence of any new bands but it is evident that there is broadening in both Amide I and Amide II FTIR bands. The broadening of the Amide I band may result from the formation of a more β-sheet-like structure since at the tails of the Amide I band lie the β-sheet band subcomponents [30]. Likewise, the minimum between Amide I and Amide II shows significant changes, and more evidently, there is also loss in the intensity of the Amide II band. This is confirmed by the second derivative spectra that reveal also a downshift of 6 cm^-1^ in the Amide II region (Fig 5C and 5D). This observation suggests that there is also modification of the protein Amide II band. There is also a similar downshift of 5 cm^-1^ in the Amide I frequency. Amide I and II frequencies are often affected by the strength of hydrogen bonds involving amide C = O and N-H groups. In contrast with hemoglobin, there is also no significant change in the 1396 cm^-1^ band. This may relate to the fact that there are no subunit contact sites in myoglobin and therefore no alteration in the subunit organization of the protein.

The different behavior of myoglobin versus hemoglobin glycation is interesting to note since it was previously highlighted by a comparative study on the structural stability of myoglobin and glycomyoglobin [33]. The molecular dynamics simulation study revealed an increased stability in the glycomyoglobin molecule as a consequence of increased contacts with water molecules. Moreover, another study has shown that glycation of apomyoglobin with glucose would not effect in fibril formation [34]. This stresses out the importance of the heme component in the whole glycation process. A recent molecular dynamics simulation investigation has pointed out that glucose molecules can interact with heme via the two propionate groups and aspartic and glutamic acid residues by hydrogen bonds, as well as with surrounding water molecules [35].

It was previously suggested that the coordination of distal histidine to the iron produces a scissoring motion in helices E and F which results in modifications in the tertiary structure of the tetramer, mainly in the αβ interface [36]. Raman spectroscopy studies have indicated that this scissor-like motion of helices E and F is critical in the transition from the R to the T state [37–38]. An interesting fact is that in models with mammalian (horse) and Antarctic fish hemoglobins is that the EF fragment modifications are related to a heme sliding motion that exposes the heme to a more solvent-exposed position [16–17]. This results in a shift of the heme group and a narrowing of the heme pocket as the E- and F-helices move toward each other. It is worth mentioning that these movements did not occur in a mutant myoglobin variant examining the function of the distal His [39].

Effector molecules can bind to liganded Hb and this process provides insight into Hb allosteric transition [40]. Previous studies have examined the interaction of polyphenols with serum proteins [41–42] and demonstrated that binding of these molecules to hemoglobin indicate that hydrogen-bonded interactions provide a means of transport of these substances in the blood. The results of protein glycation often vary according to the nature of the modified protein and also the type of carbohydrate molecule. Even though glucose plays the primary role in the formation of glycation products, other monosaccharides have been shown to be sufficient and even act more effectively than glucose to produce glycated variants. Among these, fructose has been shown to perform the glycation process at a much faster rate [43]. Here, we have described the effect of glucose and fructose derivatives and have shown that these also mediate structural alterations on hemoglobin and myoglobin.

### Conclusions

There seems to be a link between hemichrome formation and loss of protein functionality [44–45]. Certain reactive molecules, such as glyoxal and methylglyoxal (MG) have been shown to react with proteins to form advanced glycation end products (AGEs) following Maillard-like reaction [46]. Methylglyoxal modification enhanced the structural stability of hemoglobin but it lowered its iron-mediated oxidation reactions [47]. We suggest that tetrameric Hbs form a partial hemichrome state upon addition of MRP compounds as observed in the present spectroscopic investigations. On the same line, these Maillard reaction species act as external ligands binding to an external protein site triggering the formation of six-coordinated bis-histidyl hemichrome species. Bis-histidyl adducts seem to form not only in hemoglobin but also in a monomeric protein such as myoglobin. We have observed these effects in near physiological conditions and these events were not induced in any way by changing experimental conditions to extremes (e.g. temperature, pH, etc.). The heterogeneous and diverse nature of AGE structures often makes the application of a universal biomarker method difficult since these compounds may be present as mixtures in human serum. When examining analytical methods, the effort is to optimize quantitative methods for AGE quantification, such as HbA1c, but not for qualitative analysis. Further investigations could include more detailed studies on exact binding sites and spectroscopic determination of other food-derived compounds with hemoglobin and myoglobin.

## References

[1] Stadler RH, Blank I, Varga N, Robert F, Hau J, Guy PA, Robert MC, Riediker S. Food chemistry: acrylamide from Maillard reaction products. Nature. 2002 10 3;419(6906):449–50. doi: 10.1038/419449a 12368845 10.1038/419449a

[2] Zyzak DV, Sanders RA, Stojanovic M, Tallmadge DH, Eberhart BL, Ewald DK, et al Acrylamide formation mechanism in heated foods. J Agric Food Chem. 2003;51(16):4782–4787. doi: 10.1021/jf034180i 14705913 10.1021/jf034180i

[3] Amrein TM, Andres L, Manzardo GG, Amadò R. Investigations on the promoting effect of ammonium hydrogencarbonate on the formation of acrylamide in model systems. J Agric Food Chem. 2006 12 27;54(26):10253–61. doi: 10.1021/jf0625860 17177568 10.1021/jf0625860

[4] Stadler RH, Robert F, Riediker S, Varga N, Davidek T, Devaud S, et al In-depth mechanistic study on the formation of acrylamide and other vinylogous compounds by the Maillard reaction. J Agric Food Chem. 2004 8 25;52(17):5550–8. doi: 10.1021/jf0495486 15315399 10.1021/jf0495486

[5] Hidalgo FJ, Delgado RM, Navarro JL, Zamora R. Asparagine decarboxylation by lipid oxidation products in model systems. J Agric Food Chem. 2010 9 9;58(19):10512–7. doi: 10.1021/jf102026c 20828127 10.1021/jf102026c

[6] Srikanth V, Maczurek A, Phan T, Steele M, Westcott B, Juskiw D, Münch G. Advanced glycation endproducts and their receptor RAGE in Alzheimer's disease. Neurobiol. aging. 2011 5 31;32(5):763–77. doi: 10.1016/j.neurobiolaging.2009.04.016 19464758 10.1016/j.neurobiolaging.2009.04.016

[7] Gul A, Rahman MA, Salim A, Simjee SU. Advanced glycation end products in senile diabetic and nondiabetic patients with cataract. J Diab Complications. 2009;23(5):343–348. 10.1016/j.jdiacomp.2008.04.001 18508288

[8] Deli G, Bosnyak E, Pusch G, Komoly S, Feher G. Diabetic neuropathies: diagnosis and management. J Neuroendocrinol. 2013;98(4):267–280. 10.1159/000358728 24458095

[9] Paneni F, Beckman JA, Creager MA, Cosentino F. Diabetes and vascular disease: pathophysiology, clinical consequences, and medical therapy: part I. Eur Heart J. 2013;34(31):2436–2443. doi: 10.1093/eurheartj/eht149 23641007 10.1093/eurheartj/eht149 PMC3743069

[10] Nass N, Bartling B, Santos AN, Scheubel RJ, Börgermann J, Silber RE, et al Advanced glycation end products, diabetes and ageing. Z Gerontol Geriat. 2007 10 1;40(5), 349. 10.1007/s00391-007-0484-9 17943238

[11] Little RR, Rohlfing CL. The long and winding road to optimal HbA1c measurement. Clinica Chimica Acta. 2013 3 15;418:63–71. 10.1016/j.cca.2012.12.026 PMC4762213 23318564

[12] Pan T, Li M, Chen J, Xue H. Quantification of glycated hemoglobin indicator HbA1c through near-infrared spectroscopy. J Innov Opt Health Sci. 2014 7;7(04):1350060.

[13] Shapiro R, McManus MJ, Zalut C, Bunn HF. Sites of nonenzymatic glycosylation of human hemoglobin A. J Biol Chem. 1980 4 10;255(7):3120–3127. 7358733

[14] Ahmed N, Dobler D, Dean M, Thornalley PJ. Peptide mapping identifies hotspot site of modification in human serum albumin by methylglyoxal involved in ligand binding and esterase activity. J Biol Chem. 2005 2 18;280(7):5724–32. doi: 10.1074/jbc.M410973200 15557329 10.1074/jbc.M410973200

[15] Sugawara Y, Kadono E, Suzuki A, Yukuta Y, Shibasaki Y, Nishimura N, et al Hemichrome formation observed in human haemoglobin A under various buffer conditions. Acta Physiol Scand. 2003 9 1;179(1):49–59. doi: 10.1046/j.1365-201X.2003.01142.x 12940938 10.1046/j.1365-201X.2003.01142.x

[16] Vergara A, Vitagliano L, Di Prisco G, Verde C, Mazzarella L. Spectroscopic and crystallographic characterization of hemichromes in tetrameric hemoglobins. Methods Enzymol. 2008 12 31;436A:421–440. 10.1016/S0076-6879(08)36024-8 18237647

[17] Robinson VL, Smith BB, Arnone A. A pH-dependent aquomet-to-hemichrome transition in crystalline horse methemoglobin. Biochem. 2003 9 2;42(34):10113–10125. 12939139 10.1021/bi030059t

[18] Ioannou A, Daskalakis V, Varotsis C. Detection of Maillard reaction products by a coupled HPLC-Fraction collector technique and FTIR characterization of Cu (II)-complexation with the isolated species. J Mol Struct. 2017 8 5;1141:634–642.

[19] Ioannou A, Varotsis C. Real time monitoring the Maillard reaction intermediates by HPLC-FTIR. J. Phys. Chem. Biophys. 2016;6:210.

[20] Yaylayan VA, Wnorowski A, Perez Locas C. Why asparagine needs carbohydrates to generate acrylamide. J Agric Food Chem. 2003 3 12;51(6):1753–1757. doi: 10.1021/jf0261506 12617619 10.1021/jf0261506

[21] Ajandouz EH, Tchiakpe LS, Ore FD, Benajiba A, Puigserver A. Effects of pH on Caramelization and Maillard Reaction Kinetics in Fructose-Lysine Model Systems. J Food Sci. 2001 9 1;66(7):926–931.

[22] Benjakul S, Lertittikul W, Bauer F. Antioxidant activity of Maillard reaction products from a porcine plasma protein–sugar model system. Food Chem. 2005 11 30;93(2):189–196.

[23] Lertittikul W, Benjakul S, Tanaka M. Characteristics and antioxidative activity of Maillard reaction products from a porcine plasma protein–glucose model system as influenced by pH. Food Chem. 2007 12 31;100(2):669–677.

[24] Sugiyama K, Sakai T, inventors; Arkray, Inc., assignee. Method of measuring glycated hemoglobin concentration. United States patent US 8,021,887. 2011 Sep 20.

[25] Litvinko NM, Andreiuk GM, Kisel MA, Kiselev PA & Akhrem AA. The use of hemoglobin for determination of phospholipase A2 activity. Prikl Biokhim Mikrobiol. 1989;25:847–852. 2631109

[26] Dousseau F, Pezolet M. Determination of the secondary structure content of proteins in aqueous solutions from their amide I and amide II infrared bands. Comparison between classical and partial least-squares methods. Biochem. 1990 9;29(37):8771–9. 2271555 10.1021/bi00489a038

[27] Bath A, Zscherp C. What vibrations tell about proteins. Q. Rev. Biophys. 2002;35:369–430. 12621861 10.1017/s0033583502003815

[28] Bandekar J. Amide modes and protein conformation. Biochim Biophys Acta. 1992 4 8;1120(2):123–143. 1373323 10.1016/0167-4838(92)90261-b

[29] Krimm S, Bandekar J. Vibrational spectroscopy and conformation of peptides, polypeptides, and proteins. Adv Protein Chem. 1986 12 31;38:181–364. 3541539 10.1016/s0065-3233(08)60528-8

[30] Garidel P, Schott H. Fourier-transform midinfrared spectroscopy for analysis and screening of liquid protein formulations. BioProcess Int. 2006 6;4(6):48–55.

[31] Litvinov RI, Faizullin DA, Zuev YF, Weisel JW. The α-helix to β-sheet transition in stretched and compressed hydrated fibrin clots. Biophys J. 2012 9 5;103(5):1020–1027. doi: 10.1016/j.bpj.2012.07.046 23009851 10.1016/j.bpj.2012.07.046 PMC3433599

[32] Baldwin J, Chothia C. Haemoglobin: the structural changes related to ligand binding and its allosteric mechanism. J Mol Biol. 1979 4 5;129(2):175–220. 39173 10.1016/0022-2836(79)90277-8

[33] Alizadeh-Rahrovi J, Shayesteh A, Ebrahim-Habibi A. Structural stability of myoglobin and glycomyoglobin: a comparative molecular dynamics simulation study. J Biol Phys. 2015 9 1;41(4):349–366. doi: 10.1007/s10867-015-9383-2 25701404 10.1007/s10867-015-9383-2 PMC4550620

[34] Iannuzzi C, Maritato R, Irace G, Sirangelo I. Glycation accelerates fibrillization of the amyloidogenic W7FW14F apomyoglobin. PLoS One. 2013 12 4;8(12):e80768 doi: 10.1371/journal.pone.0080768 24324625 10.1371/journal.pone.0080768 PMC3851467

[35] You Y, Liu F, Du KJ, Wen GB, Lin YW. Structural and functional alterations of myoglobin by glucose-protein interactions. J Mol Model. 2014 7 1;20(7):2358 doi: 10.1007/s00894-014-2358-6 24990797 10.1007/s00894-014-2358-6

[36] Riccio A, Vitagliano L, di Prisco G, Zagari A, Mazzarella L. The crystal structure of a tetrameric hemoglobin in a partial hemichrome state. Proc Natl Acad Sci. 2002 7 23;99(15):9801–9806. doi: 10.1073/pnas.132182099 12093902 10.1073/pnas.132182099 PMC125021

[37] Rodgers KR, Spiro TG. Nanosecond dynamics of the R-T transition in hemoglobin: ultraviolet Raman studies. Science. 1994 9 16;265(5179):1697 8085153 10.1126/science.8085153

[38] Jayaraman V, Rodgers KR, Mukerji I, Spiro TG. Hemoglobin allostery: resonance Raman spectroscopy of kinetic intermediates. Science. 1995 9 29;269(5232):1843–1848. 7569921 10.1126/science.7569921

[39] Dou Y, Admiraal SJ, Ikeda-Saito M, Krzywda S, Wilkinson AJ, Li T, Olson JS, Prince RC, Pickering IJ, George GN. Alteration of Axial Coordination by Protein Engineering in Myoglobin BISIMIDAZOLE LIGATION IN THE His^64^ → Val/Val^68^ → His DOUBLE MUTANT. J Biol Chem. 1995 7 7;270(27):15993–16001. 7608158 10.1074/jbc.270.27.15993

[40] Chen Q, Lalezari I, Nagel RL, Hirsch RE. Liganded hemoglobin structural perturbations by the allosteric effector L35. Biophys J. 2005 3 31;88(3):2057–2067. doi: 10.1529/biophysj.104.046136 15626716 10.1529/biophysj.104.046136 PMC1305258

[41] Lu Z, Zhang Y, Liu H, Yuan J, Zheng Z, Zou G. Transport of a cancer chemopreventive polyphenol, resveratrol: interaction with serum albumin and hemoglobin. J fluoresc. 2007 9 1;17(5):580–7. doi: 10.1007/s10895-007-0220-2 17597382 10.1007/s10895-007-0220-2

[42] Hegde AH, Sandhya B, Seetharamappa J. Investigations to reveal the nature of interactions of human hemoglobin with curcumin using optical techniques. Int J Biol Macromolec. 2013 1 31;52:133–8. 10.1016/j.ijbiomac.2012.09.015 23022269

[43] Schalkwijk CG, Stehouwer CD, van Hinsbergh VW. Fructose-mediated non-enzymatic glycation: sweet coupling or bad modification. Diabetes Metab Res Rev. 2004 9 1;20(5):369–382. doi: 10.1002/dmrr.488 15343583 10.1002/dmrr.488

[44] Banerjee S, Maity S, Chakraborti AS. Methylglyoxal-induced modification causes aggregation of myoglobin. *Spectrochim*. Acta Part A: Mol Biomol Spectrosc. 2016 2 15;155:1–10. 10.1016/j.saa.2015.10.022 26554310

[45] Sen S, Bose T, Roy A, Chakraborti AS. Effect of non-enzymatic glycation on esterase activities of hemoglobin and myoglobin. Mol Cell Biochem. 2007 7 1;301(1–2):251–257. doi: 10.1007/s11010-007-9418-5 17549609 10.1007/s11010-007-9418-5

[46] Banerjee S, Chakraborti AS. Structural alterations of hemoglobin and myoglobin by glyoxal: a comparative study. Int J Biol Macromolec. 2014 5 31;66:311–318. 10.1016/j.ijbiomac.2014.02.034 24613676

[47] Banerjee S, Chakraborti AS. Methylglyoxal modification enhances the stability of hemoglobin and lowers its iron-mediated oxidation reactions: An in vitro study. Int J Biol Macromolec. 2017 2 28;95:1159–1168. 10.1016/j.ijbiomac.2016.11.006 27825993

